# Two decades ago, giant viruses were discovered: the fall of an old paradigm

**DOI:** 10.3389/fmicb.2024.1356711

**Published:** 2024-02-23

**Authors:** Diego Simón, Natalia Ramos, Guillermo Lamolle, Héctor Musto

**Affiliations:** ^1^Laboratorio de Genómica Evolutiva, Departamento de Biología Celular y Molecular, Facultad de Ciencias, Montevideo, Uruguay; ^2^Laboratorio de Virología Molecular, Facultad de Ciencias, Centro de Investigaciones Nucleares, Universidad de la República, Montevideo, Uruguay; ^3^Laboratorio de Evolución Experimental de Virus, Institut Pasteur de Montevideo, Montevideo, Uruguay; ^4^Sección Virología, Departamento de Biología Celular y Molecular, Instituto de Biología, Facultad de Ciencias, Universidad de la República, Montevideo, Uruguay

**Keywords:** giant viruses, Mimivirus, horizontal gene transfer, viral evolution, viral size limits

## Introduction

Arguably, one of the most exciting moments in science, and definitely in molecular biology, is when a well-established paradigm is overturned [we use the concept “paradigm” as defined by Kuhn (1962)]. Notable cases include, for example, the discovery that DNA (and not proteins) as the genetic material (Avery et al., 1944), which was later confirmed by Hershey and Chase (1952); the existence of mobile genetic elements (McClintock, 1950, 1953); the isolation of reverse transcriptases, which make DNA from an RNA template (Baltimore, 1970; Temin and Mizutani, 1970); the finding that genes in eukaryotes (and DNA viruses that replicate in eukaryotes) have “intervening sequences,” that is, they are not continuous as are the vast majority of prokaryotic genes (Berget et al., 1977; Chow et al., 1977; Jeffreys and Flavell, 1977). Perhaps needless to say, the regions that remain in the mature RNA were named exons and the “intervening sequences” introns in a brilliant paper written by Gilbert (1978) a few months after this discovery. A final example, arbitrarily chosen, is the experimental discovery that RNA (and not only proteins) can act as enzymes: the ribozymes (Kruger et al., 1982). These RNA catalysts turned out to be very important in the discussion of the origin of life –but will not be discussed further.

Certainly, there are several other discoveries that have changed our view of molecular biology and molecular evolution –for reasons of space we shall not mention them, but to the best of our knowledge, they have not changed what we usually consider as paradigms.

## Awakening “giants”

According to our opinion, one of the last “shocks” to our accepted paradigms occurred two decades ago, and it is related to viruses. Until 2003, viruses were defined as biological entities that are obligate intracellular parasites. That is, they must replicate within living organisms (Bacteria, Archaea or Eukaryotes), since (a) they lack an independent way of obtaining energy, and (b) they cannot by themselves make their own proteins since they always lack the genes coding for independent ribosomes, aminoacyl t-RNA synthetases, elongation factors, etc. A third crucial aspect of viruses, is that they were cataloged during more than a century as tiny (in relation to free-living organisms), since the virions were considered to range in size from around 20 nm (*Parvoviridae*) to 300 nm (*Poxviridae*). Given this assumption, one of the most used methods for isolating viruses was (and still is) to use filters with pores typically of 200 nm to a maximum of 450 nm. Therefore, all living cells are retained by the filter and the only entities that go through the pores are, by this technical approach, smaller than 200–450 nm. We should stress that this technique was very useful and logical, since although there are prokaryotes as small as 250 nm (for example, *Mycoplasma* spp.), and therefore can pass through the pores, the vast majority are much greater and retained by the filter; and needless to say, eukaryotic cells are much greater with sizes that are generally in the range of 10^4^ to 10^5^ nm. As a consequence, it was universally accepted that although some exceptional prokaryotes can be very tiny, falling in the range of the biggest virions, viruses were always smaller than 300 nm. In other words, this size of viruses became a paradigm, and all of us who studied virology in the last century accepted this limited size of viruses as a dogma.

But this view changed dramatically two decades ago. Indeed, in a paper published in 2003, a giant virus was isolated from the amoebae *Acanthamoeba polyphaga* (La Scola et al., 2003). They have an icosahedral capsid with a diameter of 500 nm, with an overall virion diameter of ~750 nm when including the surface fibers (Xiao et al., 2005). This size exceeded the smallest Bacteria and Archaea, and was named Mimivirus (“mimicking microbes”). But the surprise was not only by the size of the virions, yet the size of the genetic material: it was a double-stranded DNA with a length of 1.2 Mb (Raoult et al., 2004), which again exceeded the size of the smallest parasitic and even free-living Bacteria and Archaea. But perhaps most intriguingly, at the time its genome encoded genes not present in other nucleocytoplasmic large DNA viruses, or NCLDVs (Raoult et al., 2004; Iyer et al., 2006).

The discovery of the first giant virus prompted several groups to isolate and sequence other similar viruses. And surprisingly, it was found that some genomes of these entities, code for genes previously found only in free-living organisms: t-RNAs, aminoacyl-tRNA synthetases, translation factors, nucleotide synthesis, amino acid metabolism, protein modification, lipid or polysaccharide metabolism, DNA repair or protein folding (Raoult et al., 2004). These genes are supposed to have been acquired by events of horizontal gene transfer. Acquiring these genes/pathways may allow them not to be totally dependent on their hosts, thus allowing opposing compositional patterns to their hosts (Simón et al., 2021).

The origin and evolution of these viruses are still under debate and no theory can be ruled out (Abrahão et al., 2017; Koonin and Yutin, 2018). In our opinion, giant viruses arose from smaller viruses through horizontal transfer events from their host cells, and gene duplication events (see for example: Yutin et al., 2014; Machado et al., 2023). Regardless of their origin, giant viruses could be very ancient, possibly coexisting with the earliest eukaryotes. Evidence for this is that members of the NCLDVs could infect such disparate hosts, even though they show differences of up to an order of magnitude in genome size (Yutin et al., 2014). Furthermore, since some amoebae have disproportionately large genomes (up to 100 or 200 times in size vs. human), this may indicate that hosts and viruses are being subjected to the same mechanisms or selective pressures.

## Discussion

The diversity of giant viruses we know today is much greater. Besides amoebas (for a review see Aherfi et al., 2016), other protists do serve as giant virus reservoirs. Viruses exhibiting genomes larger than 1 Mb had been found in algae (Blanc-Mathieu et al., 2021) and kinetoplastids (Deeg et al., 2018). Recently-established viral class *Megaviricetes* (see ICTV Master Species List #38 at https://ictv.global/taxonomy/) includes (up to now) 9 different viral families: *Allomimiviridae, Ascoviridae, Iridoviridae, Mamonoviridae, Marseilleviridae, Mesomimiviridae, Mimiviridae, Phycodnaviridae*, and *Schizomimiviridae*. Outside this classification are the members of genera *Pandoravirus* (Philippe et al., 2013) and *Pithovirus* (Legendre et al., 2014). Moreover, advances in metagenomics have broadened the knowledge about other giant viruses, even for unknown hosts (Kristensen et al., 2010; Schulz et al., 2022). These advances have also led to the discovery of “virophages,” or viruses that parasitize giant viruses (La Scola et al., 2008). All known virophages are grouped into the family *Lavidaviridae* (Krupovic et al., 2016).

The discovery and study of these entities has opened a new frontier in microbiology. Indeed, the above mentioned findings were not only surprising, but changed our opinion about the evolution of these viruses. Some open questions are (1) since the majority of putative genes are Orphans, how do these appear and evolve? Are they functional? (2) These viruses evolved by the reduction of genes from a common free-living ancestor, or they are the result of a virus (or viruses) that acquired genes mainly by horizontal gene transfer (as generally assumed) and duplication? (3) Where are the origins of replications located? (4) Which is the maximum size of a virion? (5) Which is the maximum length of DNA in viruses?

As can be seen in this communication, when a paradigm falls, new and crucial questions arise, in this case, for people working in (but not limited to) virology and molecular evolution. A paradigm has fallen… and the field is in motion. Going forward, the journey ahead holds much to learn about giant viruses.

## Author contributions

DS: Writing – review & editing. NR: Writing – review & editing. GL: Writing – original draft. HM: Writing – original draft.
